# Not All Sleep Loss Is Equal: A Comprehensive Evaluation of Rodent Models, Their Neurobiological Validity, and Translational Relevance to Neurological Disease

**DOI:** 10.3390/biomedicines14061376

**Published:** 2026-06-18

**Authors:** Edem Ekpenyong Edem, Sabiu Bala Soja, Mohammed Rabiu Abba, Kelechi Favour Chinyere, Linus Anderson Enye

**Affiliations:** 1 Department of Human Anatomy, College of Medicine and Health Sciences, Afe Babalola University, Ado-Ekiti P.M.B. 5454, Ekiti, Nigeria; sabiu.soja@fuhsa.edu.ng (S.B.S.); mohammed.abba@fuhsa.edu.ng (M.R.A.); favourchinyere588@gmail.com (K.F.C.); enyelinus@abuad.edu.ng (L.A.E.); 2 Stress & Neuroimmunology Group, Department of Human Anatomy, College of Medicine and Health Sciences, Afe Babalola University, Ado-Ekiti P.M.B. 5454, Ekiti, Nigeria; 3 Department of Human Anatomy, Faculty of Basic Medical Sciences, Federal University of Health Sciences, Azaire 751101, Bauchi State, Nigeria

**Keywords:** sleep deprivation, rodent models, neurodegeneration, translational neuroscience, HPA axis, neuroinflammation

## Abstract

Not all sleep loss is equal, and overlooking this limits progress in sleep and neurological disease research. We compared nine rodent sleep deprivation paradigms, gentle handling, multiple platform variants, disk-over-water, the Unpredictable Chronic Sleep Deprivation (UCSD) paradigm, novel object introduction, curling prevention by water, automated systems, and head-lifting, evaluating stress confounds, sleep stage specificity, chronicity, and neurobiological outcomes. Effects included hippocampal plasticity, prefrontal chemistry, glymphatic clearance, neuroinflammation, oxidative stress, neurogenesis, and circadian regulation, linked to Alzheimer’s, Parkinson’s, and psychiatric comorbidities. UCSD with caffeine produced antioxidant depletion, serotonin reduction, acetylcholinesterase upregulation, and synaptophysin loss, early neurodegeneration markers. We propose a disease-targeted framework with six translational priorities and reporting standards.

## 1. Introduction

The biological necessity of sleep has been recognised throughout the history of medicine, yet only within the past three decades has it become possible to describe, with mechanistic precision, what the sleeping brain is actually doing and what is lost when it does not sleep. Sleep is not a passive state of neural quiescence. It is an active, highly organised biological process during which the brain engages in memory consolidation, synaptic homeostasis [1], metabolic waste clearance [2], neuroimmune regulation [3], and the maintenance of hormonal rhythms that govern virtually every organ system in the body. Sleep loss, whether total or partial, acute or chronic, REM-selective or broadly distributed across sleep stages, disrupts these processes in distinct ways that are neither equivalent nor interchangeable. Importantly, such disruptions are now understood at the molecular level, with mechanisms characterized in recent studies [1,2,3].

The epidemiological case for sleep as a modifiable risk factor in neurological disease is now substantial. Prospective studies involving hundreds of thousands of participants have established associations between habitual short sleep duration and increased risk of Alzheimer’s disease, Parkinson’s disease, vascular dementia, and stroke. A meta-analysis of longitudinal studies including 246,786 participants at baseline and 25,847 dementia cases concluded that sleep disturbances significantly predicted the risk of incident dementia [4]. Sleep disturbances commonly predate the onset of other hallmark clinical symptoms of Parkinson’s disease, and REM sleep behaviour disorder in particular is now recognised as one of the strongest prodromal markers of synucleinopathy, appearing years before motor symptoms in a significant proportion of patients [5]. Over a third of adults globally report sleeping fewer than seven hours per night, and in specific high-burden populations such as medical students, shift workers, and caregivers, the prevalence of chronic partial sleep restriction is substantially higher. In medical schools in sub-Saharan Africa, documented average nightly sleep durations below 5.74 h place large cohorts of future healthcare professionals in a state of chronic neurobiological impairment at precisely the stage of life when neurochemical patterns most relevant to long-term disease risk are being established [6].

The question of mechanism demands experimental models. The human brain cannot be easily interrogated at the cellular and molecular level in vivo, and the longitudinal time scales of neurodegeneration make direct experimental manipulation impractical in humans. The laboratory rodent, therefore, remains indispensable. The problem is that not all rodent sleep deprivation models are the same, and the field has not always been sufficiently attentive to this. A study using the multiple small platform method in rats, which selectively disrupts REM sleep but introduces significant stress confounds and forced immobility, is not directly comparable to a study using gentle handling for total sleep deprivation, which minimises stress but requires intensive human labour and is impractical for chronic protocols. A study examining the effects of six hours of daily sleep restriction over four weeks is not examining the same neurobiological process as a study in which animals are totally deprived for 72 h. Yet the literature frequently aggregates these findings as though they were drawn from a common experimental substrate, producing apparent contradictions that are often methodological rather than biological in origin.

A 2025 systematic review and meta-analysis of more than one hundred rodent studies examining the effects of sleep deprivation on synaptic proteins and cognitive outcomes found that the sleep deprivation (SD) paradigm was the primary driver of heterogeneity in outcome across studies, exceeding the contributions of rodent strain, sex, and brain region combined [3]. This is a striking finding. It means that the most influential variable in this body of literature is a methodological choice rather than a biological one. The field is not converging on biological truths; in significant part, it is generating an artefact of inconsistent methods applied to the same question without adequate acknowledgement of their non-equivalence.

This review takes a direct position on this problem: not all sleep loss is equal, and the failure to treat it as such is the single most important barrier to translational progress in the field. We argue that model selection must be determined by the research question, the disease target, and the specific neurobiological pathway under investigation; that the stress profile of each paradigm must be systematically characterised rather than acknowledged and set aside; and that reporting standards across the field must become more precise about what kind of sleep loss is being studied. To these ends, we systematically compare the principal rodent sleep deprivation methods, evaluate their neurobiological outcomes and translational validity, describe the design and findings of the Unpredictable Chronic Sleep Deprivation paradigm (UCSD) developed in our laboratory, and propose both a disease-target-driven model selection framework and a six-priority research agenda for the field.

## 2. Rodent Sleep Physiology: What Is Being Modelled and What Is Not

Any evaluation of rodent sleep deprivation models must begin with an honest account of the ways in which rodent sleep biology resembles and departs from that of humans. Rats and mice are polyphasic sleepers. They sleep in multiple short bouts distributed across the full 24 h cycle, with the majority of sleep concentrated in the light phase when they are behaviourally inactive, in contrast to the consolidated monophasic nocturnal sleep of humans. The average sleep bout duration in rats is approximately five to seven minutes, compared to the uninterrupted sleep cycles of 90 min duration that characterise human slow-wave and REM cycling [7]. Total sleep time in rats occupies approximately 40 to 50% of the 24 h period across light and dark phases combined, with a much higher proportion of NREM sleep relative to humans. The ratio of REM to total sleep time is broadly similar between species, but the architecture within which REM episodes occur differs substantially.

At the molecular level, however, the core architecture of sleep regulation is highly conserved. The two-process model of sleep, involving homeostatic sleep pressure (Process S) that accumulates during wakefulness and dissipates during sleep, and the circadian clock-driven Process C that gates the timing of sleep and wakefulness, operates through the same fundamental mechanisms in rodents and humans. The circadian clock is governed in both species by the suprachiasmatic nucleus (SCN) of the hypothalamus, which generates its molecular oscillation through a transcriptional-translational feedback loop involving the core clock genes CLOCK, BMAL1, PERIOD (PER1, PER2, PER3), and CRYPTOCHROME (CRY1, CRY2). The neurotransmitter systems regulating sleep-wake transitions, including glutamatergic, GABAergic, noradrenergic, serotonergic, histaminergic, and orexinergic signalling pathways, are similarly conserved across species, supporting the validity of rodent models for studying the fundamental neurochemistry of sleep loss [8].

The homeostatic drive for sleep is mediated in large part by adenosine, which accumulates in the basal forebrain and other brain regions during wakefulness and is cleared during sleep. This adenosinergic accumulation is the molecular basis of sleep pressure, and its pharmacological manipulation with caffeine, through adenosine A1 and A2A receptor antagonism, is both the world’s most widely used psychoactive intervention and one of the best-validated interactions between a dietary compound and sleep physiology [9]. The adenosine-sleep relationship is directly relevant to the UCSD paradigm and the caffeine co-treatment findings discussed later in this review, because the daily use of caffeine to suppress adenosine-driven sleepiness does not eliminate the neurobiological consequences of accumulated sleep debt; it masks their behavioural expression while the molecular damage accrues.

The glymphatic system, first characterised in mice by [2] and now increasingly validated in human studies, including a 2026 randomised crossover trial demonstrating that glymphatic clearance during normal sleep increases morning plasma levels of AD biomarkers compared to sleep deprivation [10], provides perhaps the most compelling argument for the translational value of rodent sleep research. This system uses perivascular channels along cerebral arteries, driven by aquaporin-4 (AQP4) water channels expressed on astrocytic endfeet, to facilitate convective exchange between cerebrospinal fluid and brain interstitial fluid, clearing metabolic waste products including amyloid-beta and tau. The norepinephrine oscillations that drive this clearance during NREM sleep are conserved across rodents and humans, as recently established by [11], cementing the translational relevance of rodent glymphatic models for neurodegeneration research.

It should be noted that the sleep-dependent model of glymphatic clearance, while supported by much of the current evidence, has not gone uncontested [12] reported findings using direct brain tissue tracer injection, suggesting that clearance slows during sleep and under anaesthesia rather than accelerating, a result that generated substantial methodological debate. In response, ref. [13] published a bioRxiv preprint demonstrating that glymphatic clearance is enhanced during sleep, a precursor to subsequent peer-reviewed work in 2025. This rebuttal argued that Miao’s findings were confounded by tracer injection technique and intracranial pressure artefacts. At the 2025 SLEEP Annual Meeting, a dedicated symposium debate explored whether glymphatic failure during sleep is the primary causal pathway linking sleep loss to Alzheimer’s disease pathology, or whether alternative mechanisms, including tau release driven by locus coeruleus neural firing during disrupted sleep, account for much of the observed proteinopathic burden. More broadly, reviews such as [14] highlight unresolved questions regarding the relative contributions of diffusion versus advection in interstitial transport and whether arterial pulsation or neuronal activity is the dominant driver of CSF flow. We present the glymphatic evidence here as the dominant current framework, while acknowledging that ongoing methodological debate means this framework may require revision as the field develops.

A critical caveat for all rodent sleep deprivation research concerns circadian phase. When studies are conducted during the light phase, which is the animals’ primary rest phase, they are modelling sleep loss at a time when homeostatic sleep pressure is at its nadir. This is not necessarily equivalent to the sleep loss that accumulates in night-shift workers, insomniacs, or individuals with circadian misalignment, all of whom experience sleep disruption during or around their own subjective biological night when sleep pressure is high and sleep regulatory systems are most active. Attention to the phase of sleep deprivation relative to the circadian cycle is therefore not a methodological nicety; it is a variable that can substantively alter the neurobiological outcomes observed, and it should be explicitly reported and justified in every study design.

## 3. Methods of Sleep Deprivation in Rodents: A Comprehensive Comparative Analysis

**Figure 1:**
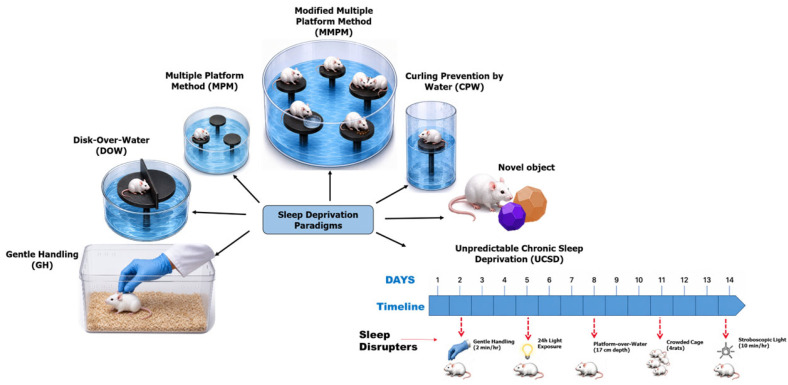
Schematic illustration of the principal sleep deprivation paradigms used in rodent studies. The figure depicts the major apparatus configurations, including the disk-over-water system, multiple small-platform paradigm, modified multiple-platform design, and cylindrical water-chamber setup. The UCSD paradigm is illustrated as a 14-day rotational disruptor timeline, emphasizing its temporal design rather than apparatus structure. Gentle handling and novel object methods are represented schematically through animal–experimenter and animal-object interactions.

The laboratory methods available for inducing sleep loss in rodents differ along several important dimensions: the type of sleep they preferentially disrupt, the degree of stress they impose alongside the deprivation, the practicality of their application over different time scales, the extent to which they can be applied to yoked control animals, and the interpretability of the data they produce. Figure 1 illustrates representative experimental setups for the principal sleep deprivation paradigms in rodents, complementing the comparative dimensions summarized in Table 1.

### 3.1. Gentle Handling

Gentle handling is the most widely used method for inducing total sleep deprivation in laboratory rodents and remains the standard against which other methods are often evaluated [15]. The procedure involves continuous monitoring of the animal, typically in its home cage, by a trained experimenter who intervenes whenever the animal displays signs of imminent sleep onset, including sustained behavioural immobility, assumption of a sleeping posture, or the appearance of low-frequency activity on electroencephalographic recordings. Intervention takes the form of gentle cage tapping, introduction of novel objects, light stroking of the animal with a soft brush, or brief handling. The objective is to maintain wakefulness with the minimum necessary level of stimulation, thereby minimising the degree of stress imposed alongside the sleep deprivation itself.

The attraction of gentle handling lies in its precision and its relatively low stress profile compared with platform-based methods. When implemented carefully, with light-phase cage tapping rather than direct handling, corticosterone levels in gently handled animals have been reported to be comparable to those of undisturbed controls [15]. This makes it possible to attribute the neurobiological outcomes observed primarily to sleep loss rather than to stress-induced glucocorticoid exposure. The method is therefore well suited to studies in which the HPA axis itself is a primary outcome variable, or in which glucocorticoid-independent effects of sleep loss on glymphatic function, synaptic plasticity, or circadian gene expression are under investigation. For studies of acute sleep deprivation lasting four to six hours, gentle handling has produced the most mechanistically granular literature currently available in sleep neuroscience, including the step-by-step characterisation of the cAMP-PKA-LIMK-cofilin signalling cascade in hippocampal CA1 neurons and the dendritic spine loss that mediates spatial memory consolidation failure [25].

The principal limitation of gentle handling is practical rather than conceptual. Maintaining truly continuous observation and intervention for periods exceeding six to eight hours requires multiple experimenters working in rotation and is extremely labour-intensive. For this reason, most gentle handling studies have been restricted to acute total sleep deprivation of four to six hours, a duration that produces reliable short-term neurobiological effects but cannot address the consequences of chronic or sub-chronic sleep loss, which are arguably more relevant to the neurodegenerative disease context. A second concern is reproducibility: the effectiveness of gentle handling varies with experimenter experience, animal temperament, and the degree of prior habituation to human contact, introducing variability that can obscure small-magnitude biological effects and that makes cross-laboratory comparisons unreliable without highly standardised protocols.

### 3.2. Platform-over-Water Methods: Multiple Small Platforms and the Inverted Flowerpot

The platform-over-water paradigm, introduced in its REM-selective form by Jouvet and colleagues in the 1960s and refined through several subsequent iterations, exploits the loss of skeletal muscle tone that characterises the transition into REM sleep. Animals are placed on small platforms or inverted flowerpots positioned above a shallow water reservoir. During wakefulness and NREM sleep, the animal can remain stationary on the platform without losing balance. Upon entering REM sleep and experiencing the accompanying muscle atonia, the animal loses postural stability and is roused by contact with or falling into the water.

The multiple small platform method extends this principle by housing several animals simultaneously in a tank containing an array of small platforms, introducing a degree of social interaction that partly mitigates the isolation stress of single-animal versions. A systematic scoping review examining 54 rat studies of sleep deprivation and oxidative stress found that 68.5% of paradoxical sleep deprivation studies employed this method, reflecting its widespread adoption in the field [26]. The method is particularly valued for its ability to selectively disrupt REM sleep over extended periods of 96 h or more, a feature that is difficult to achieve with gentle handling and that allows interrogation of REM sleep-specific functions, including memory consolidation, emotional regulation, and the maintenance of monoaminergic neurotransmitter systems [15].

The stress confounds associated with platform methods are, however, substantial and should not be minimised. Animals on small platforms experience forced immobility, exposure to an unfamiliar water environment, and inability to engage in normal resting and thermoregulatory behaviours. Corticosterone levels in platform-deprived animals are consistently elevated above those of gently handled animals and even above those of animals subjected to mild restraint stress, making it difficult to attribute neurobiological findings to sleep loss alone without carefully designed control conditions [15]. The large-platform control condition, in which animals are placed on a platform wide enough to permit sleep without the risk of water contact, is intended to control for the non-sleep aspects of the procedure, but it does not fully replicate the immobility, environmental novelty, and arousal associated with the small platform condition, meaning that the control-versus-experimental comparison remains imperfect. Studies that report neuroinflammatory cytokine elevations, oxidative stress markers, or hippocampal morphological changes after MPM deprivation should be interpreted with this caveat prominent, because glucocorticoid toxicity independently produces several of the same endpoints attributed to REM sleep loss.

The modified multiple platform method introduced variants designed to permit animals to move between platforms and to access food and water, thereby reducing some sources of physical restriction. The MMPM has been widely adopted for chronic REM deprivation studies lasting days to weeks and has been specifically applied in adolescent sleep deprivation models. Studies using the MMPM in adolescent mice have demonstrated that REM sleep deprivation impairs long-term memory in ways that persist into early adulthood, mediated at least in part through suppression of hippocampal astrocyte function, a finding with developmental implications extending well beyond the acute deprivation period [27].

### 3.3. The Disk-over-Water Method

The disk-over-water method, developed by Rechtschaffen and colleagues at the University of Chicago and described in its foundational characterisation by [28], represents the most rigorously designed approach to chronic total sleep deprivation in rats. The apparatus consists of a disk spanning a tank of shallow water, divided by a partition, with the experimental and yoked control animal each occupying one side. The disk rotates slowly and continuously, and when the experimental animal shows electroencephalographic signs of sleep onset, the disk begins to rotate, causing both animals to walk or risk falling into the water. Because the yoked control is subjected to the same physical stimulus and environmental conditions as the experimental animal but is not itself falling asleep when the stimulus occurs, the design provides the most thoughtful partial control for the non-sleep consequences of the stimulation currently available in the chronic total deprivation literature.

The major contributions of disk-over-water research have been the characterisation of the syndrome that develops during extended total sleep deprivation in rats, including weight loss despite increased food intake, hyperthermia followed by hypothermia, skin lesions, and eventual death within two to three weeks, and the demonstration that these effects are attributable to sleep loss per se rather than to the experimental stress of the procedure [29]. The yoked control design has been influential in establishing the physiological necessity of sleep in the strongest available experimental terms: the same apparatus, the same physical stimulation, the same water exposure, but only animals deprived of sleep die. The method has not, however, been successfully adapted for chronic use in mice, limiting its utility in the era of transgenic mouse models of neurodegeneration, and its EEG instrumentation requirements place it outside the reach of most laboratories.

### 3.4. The Unpredictable Chronic Sleep Deprivation (UCSD) Paradigm

Fixed-schedule chronic sleep deprivation protocols share a common methodological vulnerability that is widely acknowledged but rarely addressed: habituation. When animals are exposed to a regular, predictable sequence of sleep disruption episodes over days and weeks, their neuroendocrine and behavioural responses progressively attenuate. The corticosterone response to a predictable stressor is subject to habituation in a way that the corticosterone response to an unpredictable one is not, and this habituation means that the sleep-deprived animal in week three of a fixed chronic protocol may be experiencing a qualitatively different neurobiological state from the same animal in week one. Studies that report neurochemical profiles from fixed-schedule chronic designs may therefore be capturing the consequences of acute adaptation to a predictable disruption regime as much as the consequences of the cumulative sleep loss itself.

This problem is not unique to sleep deprivation research. It is well-established in the rodent depression literature, where the introduction of temporal unpredictability was the key innovation that transformed the chronic mild stress model from a paradigm subject to rapid habituation into the unpredictable chronic mild stress (UCMS) model, which has become the most widely used and translationally valid preclinical model of major depressive disorder. The UCMS model exposed animals to a randomised daily sequence of mild stressors, with no repetition of the same stressor on consecutive days, precisely because this unpredictability was necessary to prevent the habituation that would otherwise attenuate the depressive-like phenotype over time [30,31]. The principle is well-grounded: unpredictable and uncontrollable stressors are more harmful than predictable ones at the neurobiological level, and this is not merely a behavioural observation but a mechanistic one rooted in the different HPA activation profiles of predictable and unpredictable aversive events [32].

The Unpredictable Chronic Sleep Deprivation (UCSD) paradigm was developed in our laboratory by applying this same logic to the sleep deprivation context. For this model, animals were exposed to interchanging, unpredictable sleep disruption paradigms across a fourteen-day period. A variety of established sleep disrupters were employed: gentle handling, continuous 24 h light exposure (24/0 h light/dark cycle), platform-over-water, crowded cage conditions, and stroboscopic light. These stimulations were randomly arranged so that only one type was applied per day, with no repeat of the same disruptor on successive days. This design guaranteed that animals would receive unpredictable sleep disruption throughout the entire protocol period and would be unable to anticipate either the onset or the nature of each deprivation episode, preventing the neuroendocrine habituation that attenuates neurochemical responses in fixed-schedule designs. The integration of multiple established disruptors within a single randomised framework means that the UCSD paradigm functions as a multimodal chronic sleep disruption model in which no single confound, whether water stress, immobility, or light exposure, dominates the neurobiological signal. Each disruptor contributes to the overall sleep disruption while preventing any single stressor from driving habituation.

In the primary publication from our group using the UCSD paradigm, Long–Evans rats were exposed to chronic sleep deprivation alongside high-dose caffeine treatment to evaluate the combined neuroimmune and synaptic consequences of these co-occurring exposures, which are clinically relevant given the widespread and growing use of caffeinated beverages as wakefulness aids in chronically sleep-deprived populations [20]. The results documented significant depletion of antioxidant reserves, including superoxide dismutase, catalase, and reduced glutathione in the prefrontal cortex, a significant reduction in serotonin, upregulation of acetylcholinesterase activity, and, most notably, a marked reduction in synaptophysin protein expression detected by immunohistochemistry in the prefrontal cortex. Critically, all of these outcomes were potentiated by the co-administration of high-dose caffeine, demonstrating that the commonly used strategy of consuming caffeine to manage the alertness consequences of sleep debt does not merely mask but actively compounds the neurochemical damage. These outcomes indicate that chronic sleep deprivation of the UCSD type produces a prefrontal neurochemical profile that converges on several of the markers most consistently implicated in the early stages of neurodegeneration and neuropsychiatric disorder, including synaptophysin loss, serotonin depletion, and elevated oxidative burden.

The choice of Long–Evans rats as the experimental strain for the UCSD studies merits comment. Long–Evans rats exhibit highly differentiated outbred genetic backgrounds that produce substantial individual variability in stress reactivity and sleep architecture, making them, in some respects, more representative of the individual variation seen in human populations than inbred strains. This genetic heterogeneity means that the neurochemical effects documented under the UCSD protocol cannot be attributed to strain-specific stress sensitivity, as they would be more vulnerable to with an inbred strain known for high corticosterone reactivity. The findings from Long–Evans rats, therefore, have a degree of generalisability that is not always claimed from inbred rodent sleep deprivation data.

The UCSD paradigm draws explicitly on the conceptual framework of the UCMS model, and the parallel is instructive for understanding what the paradigm is and is not claiming. The UCMS model is not arguing that chronic depression results from any specific stressor but that the unpredictable, uncontrollable quality of chronic adversity is what produces the persistent neurobiological phenotype. Similarly, the UCSD paradigm is not arguing that the neurochemical outcomes documented reflect any one of its component disruptors; it is arguing that unpredictable, multi-modal chronic sleep disruption, regardless of the specific mechanism of each episode, produces a sustained neurobiological state of prefrontal insult that is not attenuated by the adaptation that would occur under a fixed schedule. The finding that this sustained state produces synaptophysin loss comparable in its anatomical specificity to early neurodegeneration biomarkers is not incidental; it is precisely what motivated the multi-disruptor unpredictable design.

### 3.5. Novel Object and Enriched Environment Methods

A recurring and legitimate criticism of all forced-wake paradigms is that they cannot cleanly separate the neurobiological consequences of sleep loss from those of the mechanical or social stress through which wakefulness is enforced. A fundamentally different approach exploits the naturally high arousal response of rodents to novel stimuli, inducing voluntary wakefulness through exploratory drive without requiring any restraint, water exposure, or experimenter handling. Placing novel objects in the home cage at the beginning of the sleep phase maintains wakefulness through the animal’s own behavioural motivation rather than through external compulsion. A standardised seven-day protocol using daily four-hour exposures to a curated set of novel objects was recently characterised in mice and found to minimise stress induction while effectively disrupting sleep, with serum corticosterone concentrations not increasing significantly over the seven-day period [21]. This makes the novel object method currently the closest available approximation to a truly stress-independent sleep deprivation paradigm in rodents.

The limitations of the approach are intrinsic to its mechanism. Novelty habituates. Over repeated exposures, the exploratory drive that sustains wakefulness progressively diminishes, and by the later days of extended protocols, sleep deprivation efficacy declines relative to earlier exposures. This is not simply a practical nuisance but a validity concern: if the protocol is no longer effectively depriving animals of sleep by day five of a seven-day design, then the neurobiological outcomes attributed to the final days of exposure are not sleep deprivation outcomes at all. Rotating object repertoires and carefully monitoring the sleep-wake behaviour of individual animals throughout the protocol are, therefore, necessary to maintain the validity of the method. The approach is best suited to studies of relatively short-term sleep disruption, to adolescent cohorts where HPA sensitivity makes low-stress designs essential, and to studies specifically investigating the consequences of sleep loss on glymphatic clearance, circadian clock gene expression, or neurogenesis, where glucocorticoid confounds would be most problematic.

### 3.6. The Curling Prevention by Water Paradigm

A 2023 *Cell* paper by Sang and colleagues introduced the curling prevention by water (CPW) paradigm, in which mice are housed in shallow water-filled cylinders that prevent the curling body posture associated with sleep onset, achieving approximately 96% wakefulness over the deprivation period without continuous EEG monitoring. Within four days, approximately 80% of CPW-subjected animals die, exhibiting a syndrome characterised by severe systemic inflammation, pro-inflammatory cytokine upregulation, neutrophil accumulation, and multi-organ damage affecting the liver, spleen, lung, intestine, and kidney. Immunodeficient animals lacking cytokine signalling pathways were resistant to SD-induced mortality, and pharmacological dampening of the cytokine response with acetaminophen or corticosterone conferred survival, directly implicating immune pathway activation as the proximate cause of death [22]. The corticosterone levels in CPW animals were comparable to those of animals subjected to conventional automated deprivation devices, indicating that the lethality is attributable to the sleep deprivation itself rather than to superior apparatus stress.

The CPW paradigm is of limited applicability to chronic sleep disruption or recovery-sleep research because its lethality precludes extended protocols and post-deprivation assessment. Its primary scientific contribution is the identification of the cytokine storm as the mechanistic endpoint of extreme sleep deprivation and the establishment of cytokine signalling pathways as targets for pharmacological intervention in the neuroimmune consequences of severe sleep loss. This finding is directly relevant to understanding the neuroimmune damage of chronic clinical conditions involving severe sleep disruption, and it establishes that immune pathway activation is not merely a correlate but the proximate cause of the most severe neurobiological consequences of sleep loss.

### 3.7. Automated Mechanical Systems and the Head-Lifting Method

Automated sleep deprivation systems, including motorised treadmills, rotating wheels, conveyor belts, and shaking platforms triggered by electroencephalographic detection of sleep onset, address the practical limitations of gentle handling while maintaining a degree of precision in sleep monitoring. They can be operated continuously without experimenter attendance, making them suitable for sub-chronic and chronic protocols. A shaking platform protocol validated in mice demonstrated effective and robust deprivation of both NREM and REM sleep without signs of excessive stress in adult animals [3]. However, all existing automated deprivation systems introduce forced locomotion or physical stimulation that is not purely a consequence of sleep loss, and the magnitude of this confound varies considerably across systems. Conveyor and treadmill-based systems require continuous walking that alters energy metabolism, body temperature, and corticosterone in ways that can be difficult to disentangle from the effects of the sleep loss they produce.

The head-lifting method, developed for selective REM deprivation, is a fundamentally different approach in which the beginning of each REM sleep episode is identified from polygraphic records by the experimenter in an adjacent room, who then presses a lever to gently lift the animal’s head and terminate the REM episode without direct handling or physical restraint. This method achieves 90 to 95% elimination of REM sleep with no significant reduction in NREM sleep and is considered among the lowest-stress approaches for selective REM deprivation [15]. It is, however, low throughput, requires polysomnographic expertise, and has not been adapted for chronic protocols, limiting its application to acute studies of REM-specific cognitive and neurochemical function.

## 4. Neurobiological Outcomes Across Deprivation Models

The neurobiological consequences of sleep loss span multiple systems, and their expression in any given study reflects the interaction between the biological effects of sleep loss per se and the methodological characteristics of the paradigm used to induce it. Table 2 provides a structured synthesis of the principal neurobiological outcome domains documented across rodent models, the paradigms most associated with each, the key findings, and their translational relevance to neurological disease.

### 4.1. HPA Axis Activation and Glucocorticoid Exposure

The relationship between sleep deprivation and HPA axis activation is bidirectional and method-dependent in ways that have important implications for interpreting neurobiological outcomes. Glucocorticoids affect hippocampal neurogenesis, synaptic plasticity, neuroinflammatory signalling, and gene expression across a wide range of brain regions, meaning that studies in which sleep deprivation and glucocorticoid elevation are confounded cannot easily attribute their findings to the loss of sleep per se. As a general principle, platform-based methods produce the largest and most sustained elevations in corticosterone, followed by automated mechanical methods, with gentle handling and novel object introduction producing the smallest stress hormone responses [15]. This gradient has direct implications for the neurobiological phenotypes observed.

A systematic scoping review of oxidative stress in sleep-deprived rat models found that most studies documented elevated corticosterone alongside oxidative stress markers, but that the SD-specific HPA activation profile was distinguishable from that of classical chronic stress paradigms: the corticosterone rise under SD is gradual rather than acute, the ACTH response is diminished rather than elevated, suggesting suprarenal rather than pituitary-driven release, and adrenal gland hypertrophy is absent [26]. These features suggest that sleep deprivation engages glucocorticoid pathways through mechanisms that are not fully reducible to the handling stress of the deprivation procedure, but they do not eliminate the confound; they complicate it. Studies should report corticosterone alongside sleep outcomes as a matter of course, and the SD-specific glucocorticoid signature should be distinguished from conventional stress responses rather than simply noted and set aside.

Under the UCSD paradigm, the unpredictable scheduling prevents the glucocorticoid habituation that attenuates corticosterone responses in fixed-schedule protocols, potentially sustaining the downstream consequences of HPA activation, including hippocampal dendritic atrophy, dentate gyrus neurogenesis suppression, and BDNF reduction, throughout the full fourteen-day chronic exposure period in a way that fixed designs cannot achieve after the first week. This is not merely a methodological refinement; it is the mechanism by which the UCSD paradigm models the clinically observed neurobiological profile of individuals who live with chronic unpredictable sleep disruption over months and years.

### 4.2. Glymphatic Clearance and Proteinopathic Burden

The discovery of the glymphatic system by [37] and its characterisation during sleep by [2] transformed the understanding of why sleep is necessary for brain health and introduced a mechanistic framework that is directly relevant to the pathogenesis of Alzheimer’s and Parkinson’s diseases. The glymphatic system facilitates the clearance of metabolic waste from the brain interstitium, including amyloid-beta, tau, and alpha-synuclein, through a system of perivascular channels driven by AQP4-mediated astrocytic water transport and, more recently characterised, by infraslow oscillations in norepinephrine that modulate vascular dynamics during NREM sleep [11]. These norepinephrine oscillations, which drive synchronised vascular volume changes that pump cerebrospinal fluid through the perivascular space, represent an elegant cellular mechanism linking NREM sleep quality directly to amyloid and tau clearance efficiency.

The translational evidence has now reached human clinical proof-of-concept. A 2026 randomised crossover trial in 39 human participants demonstrated that sleep-active glymphatic processes increase morning plasma levels of AD biomarkers compared to sleep deprivation, providing direct experimental evidence of glymphatic function as a modifiable target in humans and validating the translational relevance of the rodent glymphatic literature [10]. In rodent models, chronic sleep fragmentation for 30 days significantly reduced CSF tracer influx into the brain parenchyma of young wildtype mice and impaired the efflux of exogenously infused amyloid-beta from the prefrontal cortex, demonstrating that fragmented sleep, rather than total deprivation, is sufficient to compromise glymphatic function over extended time scales [38]. This finding is directly relevant to the UCSD paradigm, which produces fragmented and disrupted sleep rather than continuous total deprivation, and which therefore models the glymphatic consequences of clinical sleep disorders more faithfully than acute total deprivation designs.

In Parkinson’s disease models, sleep deprivation increased aggregates of alpha-synuclein in the brain, whereas enhancing slow-wave sleep with sodium oxybate reduced the alpha-synuclein burden, providing direct experimental evidence for the therapeutic relevance of sleep quality in synucleinopathy [5]. Most recently, acute sleep deprivation in otherwise healthy mice generated protein pathology consistent with neurodegenerative disease, including elevated oligomeric alpha-synuclein variants and TDP-43 pathology in multiple brain regions, establishing that even brief periods of sleep loss can initiate the early molecular events of neurodegeneration rather than simply accelerating a pre-existing process [8].

The method of sleep deprivation matters specifically for glymphatic research. Platform-based methods may impair glymphatic clearance through stress-induced changes in corticosterone that independently affect AQP4 expression and astrocytic morphology, rather than through the loss of the specific NREM slow-wave activity and norepinephrine oscillations that drive glymphatic flow. Studies using gentle handling or novel object introduction preserve more of the NREM architecture even while reducing total sleep time and may therefore produce different glymphatic outcomes than platform methods. This distinction should be explicitly addressed in the design and interpretation of all future glymphatic experiments.

### 4.3. Neuroinflammation and Oxidative Stress

Sleep loss promotes a pro-inflammatory state through multiple converging pathways, including microglial activation via the NF-kappaB signalling pathway, increased production of pro-inflammatory cytokines, including interleukin-1 beta (IL-1 beta), interleukin-6 (IL-6), and tumour necrosis factor-alpha (TNF-alpha), dysregulation of the NLRP3 inflammasome, and impairment of the cholinergic anti-inflammatory pathway [3]. Oxidative stress, characterised by excessive reactive oxygen species generation, lipid peroxidation, and reduced activity of antioxidant enzymes including superoxide dismutase and catalase, is consistently reported following sleep deprivation across multiple protocols and brain regions [26]. The UCSD paradigm produced significant antioxidant depletion in the prefrontal cortex of Long–Evans rats, and this depletion was potentiated by high-dose caffeine co-treatment, demonstrating that the widely used practice of using stimulants to manage the behavioural consequences of sleep debt may amplify rather than buffer the underlying oxidative neurochemical damage [20].

The relationship between sleep and cytokines is bidirectional and physiologically important. IL-1 beta and TNF-alpha are themselves sleep-promoting at physiological concentrations, suggesting that they function as homeostatic sleep pressure signals during normal wakefulness, with their pathological elevation under prolonged deprivation representing an overactivation of a system that is normally part of the regulatory machinery of sleep itself [39]. The CPW paradigm demonstrated that the cytokine response to extreme sleep deprivation can escalate to a truly pathological cytokine storm, implicating neutrophil recruitment and cytokine receptor signalling as the proximate cause of SD-induced death [22]. At sublethal levels of sleep disruption, the neuroinflammatory consequences documented in the cortex and hippocampus of chronically sleep-deprived rodents share molecular features with the profiles documented in early-stage Alzheimer’s disease and Parkinson’s disease, providing molecular mechanistic links between sleep epidemiology and neurodegeneration biology.

### 4.4. Hippocampal Neurogenesis and Synaptic Plasticity

The hippocampus is particularly vulnerable to the neurobiological consequences of sleep loss, both because it is the primary site of adult neurogenesis in the mammalian brain and because it expresses high densities of glucocorticoid receptors that render it sensitive to stress-induced damage. Sleep deprivation impairs hippocampal neurogenesis through reduced proliferation of neural precursor cells in the subgranular zone, impaired differentiation and survival of newly generated neurons, and disruption of the activity-dependent integration of new neurons into existing hippocampal circuits. A systematic review and meta-analysis of 21 rodent studies confirmed that sleep deprivation significantly reduced synaptic proteins overall, with robust cognitive impairment observed particularly in Wistar rats undergoing sleep fragmentation when assessed by the Morris water maze or novel object recognition test [3].

The mechanisms underlying sleep deprivation-induced synaptic dysfunction include reduced expression of brain-derived neurotrophic factor and its primary receptor TrkB, impaired long-term potentiation in the CA1 and CA3 regions of the hippocampus, and dysregulation of the immediate early gene Arc, which plays a central role in activity-dependent synaptic strengthening. The molecular cascade mediating LTP failure after gentle handling deprivation has been characterised with exceptional precision: reduced cAMP from elevated PDE4A5 activity leads to diminished PKA-dependent phosphorylation of LIMK, allowing cofilin to remain active and drive actin filament turnover, reducing dendritic spine density in CA1 [25]. Chemogenetic enhancement of cAMP signalling during the deprivation period rescues both spine density and LTP, providing proof-of-concept that the cAMP pathway is a pharmacologically accessible target for protecting synaptic integrity in the sleep-deprived brain [33].

### 4.5. Circadian Rhythm Disruption and Clock Gene Desynchronisation

Sleep deprivation and circadian rhythm disruption are conceptually distinct phenomena that are frequently conflated. Circadian rhythms are generated by the molecular clock mechanism in the SCN and synchronised to the external environment by light-dark cycles. Sleep is regulated by the interaction between homeostatic pressure and circadian timing but is not itself a direct output of the circadian clock. However, prolonged sleep deprivation perturbs the temporal structure of clock gene expression in multiple brain regions and peripheral organs, effectively decoupling peripheral clocks from the central SCN timing signal. The UCSD paradigm specifically incorporates continuous 24 h light exposure as one of its rotating disruptors, directly manipulating the circadian clock alongside sleep architecture in a way that models the combined circadian and sleep disruption experienced by shift workers and individuals with chronic irregular schedules.

Desynchronisation of the BMAL1 and PER1 clock genes has been documented in the context of chronic stress and gestational sleep disruption, and represents a molecular signature of HPA axis dysregulation that can outlast the period of active sleep loss. The question of whether the neurobiological damage associated with sleep deprivation is fully reversible upon recovery sleep remains inadequately answered, and studies examining the temporal recovery of clock gene expression following different types and durations of sleep loss are a priority for the field. BMAL1 loss-of-function in mice accelerates AD-relevant proteinopathy, establishing that circadian clock integrity is not merely a parallel story to sleep loss but is mechanistically linked to the neurodegeneration pathways that sleep loss also engages [8].

## 5. A Disease-Target-Driven Framework for Model Selection

The evidence reviewed in the preceding sections supports the argument that the selection of a sleep deprivation model should be driven, first and foremost, by the neurological disease target and the specific neurobiological pathway under investigation, rather than by habit, convenience, or the precedent of the published literature. Table 3 presents a structured framework for this selection process across six neurological and neuropsychiatric disease contexts.

For Alzheimer’s disease research, if the target outcome is glymphatic clearance of amyloid-beta and tau, then the method should minimally perturb residual NREM sleep architecture and should avoid glucocorticoid elevations that independently affect AQP4 expression. Gentle handling for acute deprivation and novel object introduction for sub-chronic disruption are therefore the most appropriate choices. For proteinopathic outcomes in the transgenic AD mouse literature, it is notable that APP/PS1 mice deprived of sleep by gentle handling show the specific failure of norepinephrine oscillation restoration that drives glymphatic clearance, providing a mechanistic explanation for their accelerated amyloid accumulation [34].

For Parkinson’s disease research, the central relevance is the bidirectional relationship between sleep architecture quality and alpha-synuclein burden. REM sleep behaviour disorder is an almost universal prodrome of synucleinopathy, preceding motor symptoms by up to a decade in many patients. The head-lifting method for selective REM deprivation most faithfully models this aspect of PD sleep pathology, and the sodium oxybate enhancement of SWS as a therapeutic comparator provides a pharmacological handle that no other model approach offers. The UCSD paradigm may have utility here for studying the combined effects of sleep disruption on the PD-relevant neurochemical profile, particularly the dopaminergic and serotonergic consequences of chronic unpredictable disruption.

For neuropsychiatric comorbidity research, including models of depression and anxiety that co-occur with both insomnia and neurodegenerative disease, the UCSD paradigm occupies a unique position because its prefrontal neurochemical outcomes, including serotonin depletion and synaptophysin loss, are directly relevant to the neuropathology of both MDD and the early stages of neurodegeneration. The caffeine potentiation data from the UCSD studies also position it as the model of choice for studying the clinically common but preclinically underexplored combination of chronic sleep debt and stimulant use.

## 6. Knowledge Gaps and a Structured Research Agenda

Despite the depth of the existing rodent sleep deprivation literature, several knowledge gaps represent priority targets for future research. Table 4 presents a structured six-priority research agenda, with proposed study designs and the clinical or translational significance of each priority.

The near-total absence of sex as a biological variable from the rodent sleep deprivation literature is perhaps its most glaring deficit. Female rodents show substantially different HPA axis reactivity to both sleep loss and experimental stress compared with males, driven partly by oestrogen’s modulation of corticotropin-releasing hormone expression and glucocorticoid receptor density in the paraventricular nucleus and hippocampus. The incidence of Alzheimer’s disease is consistently higher in women than in men, and the sex difference cannot be fully explained by differential survival. If female rodents respond to the same sleep deprivation protocol differently than males at the level of glymphatic clearance, HPA reactivity, and proteinopathic burden, then the field’s current male-dominated literature may be producing a systematically incomplete and potentially misleading picture of the mechanisms by which sleep loss contributes to neurological disease.

A small but informative body of studies has already included female rodents and offers preliminary evidence justifying this research priority. Ref. [40] reviewed existing sex-comparative sleep deprivation studies and identified that whereas gentle handling does not significantly elevate corticosterone in males, female rats show significantly elevated corticosterone immediately after the same procedure, driven by oestrogen’s modulation of corticotropin-releasing hormone expression. This single finding reframes the methodological assumption underlying the gentle handling low-stress designation: it may not be HPA-axis-independent in female subjects. In rats undergoing six hours of gentle handling deprivation, males showed contextual memory impairment while both sexes showed recognition memory impairment; hippocampal kynurenic acid, a tryptophan metabolite linked to cognitive dysfunction, increased only in males, and this sex-specific response was abolished by gonadectomy, establishing a direct mechanistic role for gonadal hormones in shaping the neurochemical response to sleep loss. More recently, paradoxical sleep deprivation produced anxiety-like behaviour specifically in female mice in oestrus without a corresponding locomotor increase, indicating that cycle stage mediates the behavioural expression of sleep loss in females in ways that male-only designs cannot capture. These findings are fragmentary and predominantly limited to acute gentle handling protocols; they do not yet address the consequences of the UCSD paradigm or any chronic multi-modal design in female cohorts, making the proposed parallel-cohort studies a genuine research priority rather than a replication exercise.

The question of African ecological stressors represents a specific and underexplored dimension of the sleep deprivation literature. The laboratory conditions under which essentially all rodent sleep research is conducted, thermoneutral ambient temperatures, ad libitum food and water, individual housing in standardised cages, do not model the environmental context in which sleep loss exerts its neurological effects in African populations, where gestational heat stress, food insecurity, displacement-related psychosocial adversity, and environmental toxicant exposure co-occur with disrupted sleep. Characterising the neurobiological consequences of these contextualised, ecologically valid stressors in rodent models is not merely of regional interest: the interaction between chronic environmental stress and sleep loss may produce neurobiological phenotypes that are quantitatively and qualitatively distinct from those produced by sleep loss in isolation, with implications for understanding the growing burden of neurodegeneration in African communities.

Expanding the outcome repertoire of future UCSD paradigm studies to include standardised behavioural batteries would also enable systematic comparison with non-pharmacological interventional comparators; a recent preclinical meta-analysis of eight rodent studies demonstrated that acupuncture significantly attenuated sleep-deprivation-induced cognitive deficits as assessed by Morris water maze performance, through pathways involving catecholaminergic and BDNF signalling [41] underscoring that behavioural outcomes are sensitive enough to detect therapeutic effects and should be incorporated as standard endpoints.

## 7. Discussion

The argument of this review can be stated plainly: the variety of methods used to model sleep loss in rodents produces different neurobiological outcomes because they are not modelling the same thing, and progress in translating rodent sleep findings to human neurological disease will require the field to become more precise about which aspect of sleep loss is under investigation in any given study. Sleep fragmentation, as experienced by insomniacs and patients with obstructive sleep apnoea, is biologically distinct from the total sleep deprivation induced by gentle handling, which is distinct from the REM-selective deprivation produced by platform methods, which is distinct from the chronic sleep restriction modelled by scheduled sleep opportunity reduction. These are not minor methodological variants; they engage different neurobiological mechanisms, activate the HPA axis to different degrees, produce different patterns of glymphatic impairment, and are relevant to different human disease phenotypes. The title of this review is not rhetorical.

The UCSD paradigm represents our laboratory’s direct response to the habituation problem in chronic sleep deprivation research. By randomising the sequence and type of sleep disruptors across a fourteen-day protocol with no successive repetition, the paradigm prevents the neuroendocrine adaptation that attenuates outcomes in fixed-schedule designs and produces a sustained prefrontal neurochemical profile that converges on the early neuropathological signatures of neurodegeneration. The synaptophysin loss documented in the prefrontal cortex of Long–Evans rats after UCSD exposure is particularly significant in this context. Synaptophysin is not a generic marker of cellular damage; it is a specific presynaptic vesicle protein whose reduction in the prefrontal cortex and hippocampus is one of the most consistently replicated neuropathological findings in Alzheimer’s disease, Parkinson’s disease, and schizophrenia, and whose loss correlates with cognitive performance more directly than amyloid plaque burden alone. The induction of this marker’s reduction by chronic unpredictable sleep disruption positions the UCSD paradigm within the early neuropathological trajectory of these diseases in a way that acute total deprivation models do not [20].

The glymphatic system has provided the most compelling recent advance in the mechanistic understanding of why sleep matters for neurological health. The demonstration by [11] that norepinephrine oscillations during NREM sleep drive glymphatic clearance through synchronised vascular volume changes establishes a cellular mechanism that is both elegant and clinically actionable: it identifies a specific neural process during a specific sleep stage as the driver of amyloid and tau clearance, providing a therapeutic target that is pharmacologically accessible. The subsequent demonstration in a randomised human crossover trial that sleep-active glymphatic processes increase morning plasma levels of AD biomarkers compared with sleep deprivation [10] begins to close the translational gap between the rodent glymphatic literature and the clinical reality of Alzheimer’s disease.

The implications of the caffeine co-treatment data from the UCSD paradigm deserve extended discussion because they have direct public health significance. Caffeine is the world’s most widely consumed psychoactive substance, and its use to manage the functional consequences of inadequate sleep is ubiquitous. The evidence that low-dose caffeine prevents sleep deprivation-induced LTP impairment and memory deficits through adenosine A1 receptor antagonism in acute paradigms has been widely cited as evidence for the neuroprotective potential of caffeine in the context of sleep loss. The UCSD data challenge this view substantially and specifically in the chronic context. High-dose caffeine under conditions of chronic unpredictable sleep deprivation did not attenuate neurochemical deficits; it potentiated them, producing greater antioxidant depletion, greater serotonin loss, and greater synaptophysin reduction than sleep deprivation alone. The dose dependency here is important: the protective effects of caffeine documented in acute paradigms used low doses with specific timing, while the potentiating effects documented in the UCSD paradigm involved high doses administered chronically. The practical implication is that individuals who habitually consume high quantities of caffeine to sustain functional performance across chronic sleep debt may be compounding rather than merely masking the neurological consequences of their sleep loss, and this is a message with direct public health relevance that the preclinical literature is uniquely positioned to support.

A further clinical context in which the model selection framework proposed in Table 3 has direct utility is traumatic brain injury (TBI). Sleep disruption is among the most prevalent and persistent sequelae of TBI, occurring in 30–70% of patients, and its most common manifestation is insomnia. Ref. [42] documented that fragmented or disrupted sleep following TBI worsens neuropsychiatric, behavioural, and physical symptoms and can lead to cognitive decline, with mechanisms involving damage to sleep-regulatory circuits in the hypothalamus, brainstem, and thalamus that overlap substantially with those through which chronic sleep deprivation damages the same circuits in the reverse direction. This bidirectionality means that preclinical sleep deprivation models offer a window not only into primary sleep disorders but also into the neurobiological deterioration that sleep disruption drives in post-TBI populations, and the choice of paradigm should be made with TBI-specific circuit vulnerabilities in mind.

The proposal in this review for a disease-target-driven framework for model selection is intended as a practical guide rather than a prescriptive protocol. The field will not benefit from the abandonment of any particular method: each has strengths that make it uniquely suited to specific research questions. What it will benefit from is greater explicitness about what each method models, greater rigour in the characterisation and reporting of stress profiles alongside sleep outcomes, and greater willingness to conduct direct head-to-head comparisons of methods within the same laboratory, using the same outcome measures and animal cohorts. Without such comparisons, the literature will continue to accumulate findings that are difficult to integrate and that undermine the translational case for sleep as a therapeutic target in neurological disease.

A final, necessary qualification concerns the relationship between rodent sleep deprivation models and clinical sleep disorders. These are not equivalent. Insomnia is characterised by difficulty initiating or maintaining sleep in the presence of adequate sleep opportunity, accompanied by hyperarousal, comorbid anxiety, and a dissociation between objective sleep duration and subjective sleep quality that polysomnography alone does not resolve; no forced-wake paradigm reproduces this clinical reality. Obstructive sleep apnoea involves sleep fragmentation, intermittent hypoxia, and autonomic dysregulation; current rodent models do not capture the hypoxic component, which is independently neurotoxic and confounds any attempt to isolate the consequences of sleep disruption alone. The chronic clinical trajectory of sleep disorders, unfolding across years or decades in the context of ageing, metabolic comorbidity, and shifting circadian biology, is not reproduced by any current preclinical protocol. Individual differences in sleep homeostatic capacity, which are clinically documented and partially heritable, are poorly captured in standardised laboratory cohorts. These limitations define the appropriate scope of inference from preclinical sleep deprivation research: such models provide mechanistic insight into the neurobiological pathways through which sleep loss impairs neural functions, with a degree of experimental control unavailable in human studies, but should not be presented as models of any specific clinical sleep disorder.

## 8. Conclusions

Rodent models of sleep deprivation have contributed substantially to our understanding of why sleep is necessary and what happens to the brain and body when it is lost. The neurobiological consequences of sleep loss, spanning HPA axis dysregulation, glymphatic failure, neuroinflammation, oxidative stress, impaired hippocampal neurogenesis, synaptic protein loss, and clock gene desynchronisation, are now characterised with considerable molecular precision. The UCSD paradigm developed in our laboratory adds to this literature a chronic, unpredictable, multi-modal model that prevents habituation, produces a neurochemical profile directly relevant to the early neuropathology of neurodegeneration, and generates data on the consequences of caffeine co-treatment that have direct clinical and public health implications. The challenge is not a shortage of knowledge but a shortage of integration. The evidence reviewed here demonstrates that different methods of sleep loss produce different neurobiological profiles and that the translational relevance of any given finding depends critically on which aspect of human sleep loss is being modelled. The most productive path forward involves three priorities: the adoption of method-specific reporting standards; the systematic investigation of sex and age as biological variables in sleep deprivation research; and the development of ecologically valid models that incorporate the co-stressors, including heat, dietary restriction, and psychosocial adversity, that characterise the epidemiological reality of neurological disease in diverse global populations. The sleeping brain is not merely resting. It is maintaining itself. Understanding which aspects of that maintenance are disrupted by which patterns of sleep loss, in which neurobiological systems, and with what degree of reversibility, is one of the most consequential questions in contemporary neuroscience. The rodent models reviewed here are indispensable tools for answering it, but only if they are chosen and used with the precision that the question demands.

## Figures and Tables

**Table 1:** Comprehensive comparative evaluation of nine principal rodent sleep deprivation paradigms.

Method	Sleep Stage(s) Disrupted	Stress Level	Duration Feasibility	Principal Neurobiological Outcomes	Primary Confound/Limitation	Species	Key References
Gentle Handling (GH)	Total SD (both NREM + REM)	Low to moderate; depends on direct handling vs. cage tapping	Acute (up to 8 h); chronic restriction feasible but labour-intensive	cAMP-PKA-LTP cascade disruption; hippocampal dendritic spine loss; memory consolidation failure; most mechanistically granular data in the field	Highly experimenter-dependent; inconsistent across laboratories without strict protocol; impractical for continuous multi-week chronic designs	Rat, Mouse	[15,16]
Multiple Platform Method (MPM) and modified variants	Predominantly REM; NREM partially preserved	Moderate to high; forced immobility, water aversion, and social isolation add substantially to corticosterone elevation	Sub-chronic to acute (24–96 h); modified MPM feasible for days to weeks	Spatial memory impairment; hippocampal LTP loss; neuroinflammatory cytokine elevation; neuroendocrine disruption; adolescent developmental sleep studies	Immobility and water stress confound neurochemical interpretation; large-platform control imperfectly separates SD effects from apparatus stress	Rat (primarily)	[17,18]
Disk-Over-Water (DOW)	Total SD (both stages); triggered by EEG-detected sleep onset	Moderate; yoked-control design provides best available separation of sleep-loss from apparatus stress	Chronic (weeks to months); gold standard for extended total SD; documented mortality at 2–3 weeks	Progressive weight loss, hyperthermia, immune failure, and death established as attributable to sleep loss per se; metabolic and thermoregulatory phenotypes	Requires EEG instrumentation; not adapted for mice; technically demanding; rarely reproduced outside original Rechtschaffen laboratory lineage	Rat	[19]
Unpredictable Chronic Sleep Deprivation (UCSD)	Total SD; unpredictably alternating disruptors including gentle handling, 24/0 h L/D cycle, platform-over-water, crowded cage, and stroboscopic light	Moderate; addresses habituation bias of fixed-schedule designs; stress confound profile comparable to other forced-wake paradigms	Chronic (14+ days); unpredictability prevents habituation-mediated attenuation throughout protocol	Prefrontal antioxidant depletion (SOD, catalase, GSH); serotonin loss; AChE upregulation; synaptophysin reduction by immunohistochemistry; behavioural deficits potentiated by co-administered caffeine	Systematic characterisation of unpredictability parameters needed; control animals require matched exposure variability; limited published replication data	Rat (Long–Evans)	[20]
Novel Object/Enriched Environment	Voluntary wakefulness; predominantly NREM displacement through exploratory arousal	Lowest of available methods; corticosterone does not rise significantly over a 7-day protocol	Short to sub-chronic (7-day protocol validated); habituation limits longer protocols	Adolescent sleep disruption; HPA axis responses; social and emotional behaviour; ideal when glucocorticoid confound must be minimised	Incomplete deprivation; arousal efficacy declines with repeated object exposure; object repertoire must be actively rotated	Mouse (primarily)	[21]
Curling Prevention by Water (CPW)	Total SD; approximately 96% wakefulness achieved without continuous EEG monitoring	Moderate; corticosterone comparable to other automated devices	Short-term only (up to 96 h); approximately 80% mortality by day 4 precludes chronic application	Cytokine-storm-like syndrome; neutrophil recruitment; multi-organ damage; cytokine signalling identified as proximate cause of SD-induced death	Lethality prevents chronic or recovery designs; unsuitable for studying sleep loss in the absence of extreme systemic inflammation	Mouse	[22]
Automated Treadmill/Moving Bar/Shaking Platform	Total or partial; depends on timing and EEG-coupling	Moderate; forced locomotion adds physical stress independent of sleep loss	Sub-chronic to chronic; programmable schedules enable sleep restriction designs	Cognitive function, neuroinflammation, metabolic markers; useful for sleep restriction rather than total deprivation studies	Forced locomotion confounds energy expenditure, body temperature, and musculoskeletal outcomes	Rat, Mouse	[23,24]
Head-Lifting Method	Selective REM; low NREM disruption	Low; animal remains in home cage; no water or restraint	Acute protocols validated; not adapted for chronic designs	REM-specific memory consolidation; emotional regulation; synucleinopathy-relevant REM disruption models	Requires continuous polysomnographic monitoring and trained operator; low throughput	Rat	[13]

LEGEND: SD = sleep deprivation; NREM = non-rapid eye movement sleep; REM = rapid eye movement sleep; MPM = multiple platform method; UCSD = unpredictable chronic sleep deprivation; CPW = curling prevention by water; DOW = disk-over-water; GH = gentle handling; LTP = long-term potentiation; HPA = hypothalamic–pituitary–adrenal; AChE = acetylcholinesterase; GSH = glutathione; SOD = superoxide dismutase.

**Table 2:** Neurobiological outcomes of rodent sleep deprivation and their translational relevance to neurological disease.

Outcome Domain	Brain Region/System	Deprivation Model	Key Finding	Translational Relevance to Neurological Disease	Key References
Antioxidant depletion (SOD, catalase, GSH) and AChE upregulation	Prefrontal cortex	UCSD paradigm; Long–Evans rats	Chronic SD with high-dose caffeine depleted antioxidant reserves, reduced serotonin, and elevated AChE in PFC; potentiation by caffeine exceeded SD alone	PFC oxidative stress is a shared feature of schizophrenia, MDD, and early AD/PD; UCSD model provides a controllable chronic context for studying these changes without a genetic trigger	[20]
Synaptophysin reduction	Prefrontal cortex (IHC)	UCSD paradigm; Long–Evans rats	Significant reduction in synaptophysin immunoreactivity in PFC; effect potentiated by high-dose caffeine co-treatment	Synaptophysin loss correlates with cognitive decline more reliably than amyloid plaque density in AD brains; its induction by sleep loss positions UCSD model in the early neuropathological trajectory of neurodegeneration	[20]
LTP impairment and dendritic spine loss	Hippocampal CA1; dentate gyrus	Gentle handling; modified MPM; brief 5 h deprivation	5 h SD reduces CA1 spine density; cAMP-PKA-LIMK-cofilin cascade is mechanistically necessary and sufficient; rescue of cAMP restores both LTP and spatial memory	LTP deficits and spine loss are among the earliest synaptic changes in AD transgenic models; SD offers a non-genetic, reversible approach to inducing these phenotypes acutely	[25,33]
Pro-inflammatory cytokines (TNF-α, IL-1β, IL-6) and microglial NF-κB activation	Hippocampus, cortex, systemic circulation	MPM; gentle handling; CPW paradigm; automated devices	SD elevates TNF-α and IL-1β across multiple paradigms; CPW escalates to a cytokine-storm phenotype with 80% mortality at 96 h; cytokine signalling identified as proximate lethal mechanism	Neuroinflammatory profiles at sublethal SD levels share molecular features with early AD and PD, including microglial activation, IL-1β elevation, and impaired astrocytic clearance	[3,22]
Amyloid-beta and tau accumulation; glymphatic failure	Cortex, hippocampus, CSF; perivascular channels	APP/PS1 transgenic mice; wild-type acute SD; DOW-equivalent protocols	APP/PS1 mice fail to generate norepinephrine oscillations 24 h post-SD; Abeta accumulates coinciding with impaired glymphatic clearance; one night of SD reduces Abeta clearance in healthy humans	Establishes causal rather than merely correlational link between sleep loss and AD proteinopathy; glymphatic clearance during NREM NE oscillations is a pharmacologically accessible target	[11,34,35]
Alpha-synuclein aggregation and PD-relevant proteinopathy	Nigrostriatal system; striatum	VMAT2-deficient PD mice; SWS enhancement with sodium oxybate; acute SD in wild-type	SD increases alpha-syn aggregates; slow-wave sleep enhancement with sodium oxybate reduces alpha-syn burden and improves proteostasis; TDP-43 and oligomeric alpha-syn variants elevated after acute SD	Bidirectional manipulation of sleep architecture is pharmacologically feasible in PD models; SWS-enhancing agents represent a therapeutic strategy with direct clinical trial implications	[5,8]
HPA axis dysregulation; SD-specific corticosterone profile	Hypothalamic–pituitary–adrenal axis; hippocampus; amygdala	MPM; automated devices; DOW yoked-control designs; UCSD	Elevated corticosterone near-universal across paradigms; SD-specific profile characterised by gradual rise, absent ACTH habituation, and no adrenal hypertrophy, distinguishing it from conventional chronic stress	Chronic glucocorticoid elevation drives hippocampal dendritic atrophy, impairs dentate gyrus neurogenesis, and suppresses BDNF; links sleep loss to the neurotrophic deficits of neurodegeneration through a glucocorticoid-mediated mechanism	[15,26]
Hippocampal neurogenesis impairment	Dentate gyrus subgranular zone; hippocampal CA1/CA3	MPM; gentle handling; modified MPM in adolescent mice	SD reduces proliferation and survival of neural precursor cells in SGZ; impairs activity-dependent integration of new neurons; synaptic protein loss confirmed by meta-analysis of 21 rodent studies	Hippocampal neurogenesis decline accelerates with ageing and is further impaired by the glucocorticoid profile of SD; relevant to the early cognitive decline trajectory in AD and vascular dementia	[3,36]
Circadian clock gene desynchronisation (BMAL1, PER1, PER2, CRY)	SCN; hippocampus; prefrontal cortex; peripheral organs	Chronic SD paradigms; constant-light exposure; variable light-dark manipulation	Prolonged SD decouples peripheral clocks from SCN timing; BMAL1 and PER1 desynchronisation documented after chronic stress and gestational SD; molecular recovery time course poorly characterised	Clock gene dysregulation is an independent pathway linking sleep loss to neurodegeneration; BMAL1 loss-of-function in mice accelerates AD-relevant proteinopathy; circadian misalignment is a modifiable upstream variable	[3,8]

Note. UCSD = unpredictable chronic sleep deprivation; MPM = multiple platform method; CPW = curling prevention by water; DOW = disk-over-water; PFC = prefrontal cortex; LTP = long-term potentiation; CSF = cerebrospinal fluid; HPA = hypothalamic–pituitary–adrenal; AChE = acetylcholinesterase; SOD = superoxide dismutase; GSH = glutathione; AD = Alzheimer’s disease; PD = Parkinson’s disease; MDD = major depressive disorder; SGZ = subgranular zone; SCN = suprachiasmatic nucleus; IHC = immunohistochemistry; BDNF = brain-derived neurotrophic factor.

**Table 3:** Disease-target-driven framework for rodent sleep deprivation model selection.

Disease/ Condition Target	Primary Neurobiological Outcome of Interest	Recommended Method	Rationale	Methods to Avoid and Reasons
Alzheimer’s disease	Abeta and tau accumulation; glymphatic clearance efficiency	Gentle handling (acute); novel object introduction (sub-chronic); sleep fragmentation designs	Low stress preserves interpretation of glymphatic and proteinopathic outcomes; fragmentation models insomnia and OSA phenotypes more faithfully than total SD	MPM: glucocorticoid elevation independently affects AQP4 expression and astrocytic morphology, confounding glymphatic interpretation
Parkinson’s disease and synucleinopathy	Alpha-synuclein aggregation; dopaminergic integrity; REM sleep behaviour disorder modelling	Selective REM deprivation (head-lifting); slow-wave sleep enhancement with sodium oxybate as interventional comparator	REM-specific disruption models prodromal PD sleep phenotype (RBD); SWS enhancement provides bidirectional pharmacological handle on proteinopathy	Total SD methods (GH, DOW): cannot isolate REM-specific contributions or model the selective REM architecture changes of prodromal PD
Neuropsychiatric comorbidity: depression, anxiety, schizophrenia	Prefrontal neurochemistry; serotonin; synaptophysin; antioxidant reserves; behavioural phenotype	UCSD paradigm; chronic restriction with pharmacological co-treatment	Unpredictable schedule prevents habituation; prefrontal outcomes including synaptophysin loss and serotonin depletion converge on MDD and schizophrenia neuropathology; caffeine co-treatment models clinical stimulant use	Acute GH: duration too short to produce chronic prefrontal neurochemical phenotype; novel object: insufficient depth of deprivation for neuropsychiatric modelling
Extreme sleep loss; neuroimmune lethal cascade	Cytokine storm; neutrophil biology; innate immune pathway; organ failure mechanisms	CPW paradigm; DOW-equivalent total SD for mortality end-points	CPW achieves 96% wakefulness and isolates cytokine signalling as the proximate lethal mechanism; useful for pharmacological target validation (anti-cytokine agents)	Not applicable for recovery-sleep or chronic sub-lethal designs; lethality prevents most neurological endpoints
Vascular dementia; blood-brain barrier integrity; cerebrovascular disease	Neuroinflammation; oxidative stress; BBB permeability; cerebrovascular reactivity	DOW or automated shaking (with yoked controls); chronic restriction designs	Chronic total SD models sustained cerebrovascular and metabolic stress; yoked-control DOW design separates sleep from apparatus effects	Novel object or GH: duration and depth insufficient to model the sustained vascular stress relevant to cerebrovascular disease
Developmental/transgenerational effects; adolescent neurobiology	HPA axis programming; hippocampal neurogenesis; offspring SCN-PVN axis; developmental sleep debt	Novel object (adolescent cohorts); sleep fragmentation; modified MPM for adolescent REM deprivation	Low-stress novel object protocol critical in adolescence where HPA axis is especially sensitive; fragmentation models disrupted sleep environment more realistically than total SD	DOW: not adaptable to mice used in most developmental transgenic models; full restraint and water stress contraindicated in juvenile animals

Note. SD = sleep deprivation; REM = rapid eye movement sleep; UCSD = unpredictable chronic sleep deprivation; MPM = multiple platform method; GH = gentle handling; DOW = disk-over-water; CPW = curling prevention by water; AQP4 = aquaporin-4; HPA = hypothalamic–pituitary–adrenal; BBB = blood-brain barrier; PD = Parkinson’s disease; SCN = suprachiasmatic nucleus; PVN = paraventricular nucleus; MDD = major depressive disorder.

**Table 4:** Priority knowledge gaps and a structured research agenda for rodent sleep deprivation research.

Knowledge Gap	Current State of Evidence	Proposed Study Design	Why This Matters
Sex as a biological variable in SD research	Near-total absence of female rodents in SD literature; sex differences in HPA reactivity, insomnia prevalence, and AD incidence are clinically established but preclinically uncharacterised	Parallel male and female cohorts in UCSD and GH protocols; primary outcomes: corticosterone, BDNF, synaptophysin, and glymphatic clearance metrics	AD is consistently more prevalent in women; if female rodents show different glymphatic, HPA, and synaptic responses to SD, the current male-dominated literature produces a systematically incomplete picture
Ageing as a variable in UCSD outcomes	UCSD outcomes characterised only in young adult rats; interaction between chronic unpredictable sleep disruption and the ageing brain is entirely uncharacterized	Aged rodent cohorts (18–24 months) subjected to UCSD ± caffeine; primary outcomes: synaptophysin, amyloid-beta accumulation, alpha-syn, and behavioural battery	Age is the dominant risk factor for neurodegeneration; testing whether the UCSD neurochemical profile is amplified in the aged brain would directly link the model to clinical risk populations
Glymphatic consequences of unpredictable versus fixed-schedule chronic SD	Glymphatic impairment documented after acute SD and fixed-schedule chronic fragmentation; no comparison between UCSD and fixed-schedule protocols matched for total sleep lost	Head-to-head UCSD versus fixed-schedule chronic restriction; primary outcomes: CSF tracer influx, Abeta efflux from PFC, AQP4 expression, perivascular NE oscillation amplitude	Tests whether unpredictability per se, beyond total sleep lost, produces greater glymphatic dysfunction; would establish UCSD as a model of choice for AD-relevant preclinical research
Reversibility of UCSD-induced neurochemical and synaptic outcomes	Recovery sleep outcomes after UCSD are entirely uncharacterised; unknown whether synaptophysin loss and antioxidant depletion are reversible on one-week recovery vs. persistent	Serial sampling at 24 h, 72 h, and 2 weeks post-UCSD; outcomes: synaptophysin IHC, antioxidant enzyme activity, serotonin, BDNF, BMAL1/PER1 expression	If UCSD-induced prefrontal changes persist after recovery sleep, this would establish a mechanistic basis for the clinical observation that chronically sleep-deprived individuals do not fully recover neurobiologically with brief recovery periods
African ecological co-stressors combined with SD	All published rodent SD research conducted in thermoneutral, food-secure, single-species laboratory conditions; heat stress, food insecurity, and psychosocial adversity absent from the literature	Gestational and chronic heat stress combined with UCSD; outcomes: offspring SCN-PVN programming, HPA reactivity, hippocampal neurogenesis, proteinopathic markers in aged offspring	The neurological disease burden in Africa includes environmental co-variables not captured in standard laboratory conditions; modelling ecologically valid stressors alongside SD would generate findings relevant to populations bearing the highest neurodegeneration burden
Reporting standards and minimum parameter set for SD publications	A 2025 meta-analysis found that SD paradigm was the primary driver of heterogeneity across 100+ rodent studies on synaptic proteins, exceeding contributions from strain, sex, and brain region combined	Consensus reporting guidelines: method + modifications; deprivation duration and circadian timing; sleep verification metric; strain, age, sex, housing; corticosterone at onset and endpoint; interval to outcome measurement	Without minimum reporting standards, the literature accumulates findings that cannot be synthesised, replicated, or translated; the field is generating an artefact of inconsistent methods rather than converging on biological truth

Note. SD = sleep deprivation; UCSD = unpredictable chronic sleep deprivation; IHC = immunohistochemistry; BDNF = brain-derived neurotrophic factor; AD = Alzheimer’s disease; PFC = prefrontal cortex; AQP4 = aquaporin-4; NE = norepinephrine; GH = gentle handling; HPA = hypothalamic–pituitary–adrenal; SCN = suprachiasmatic nucleus; PVN = paraventricular nucleus; BMAL1/PER = core circadian clock genes; CSF = cerebrospinal fluid.

## Data Availability

No new datasets were generated or analysed in this study. All data referenced are available in the cited published literature.
